# Elevated urine albumin-to-creatinine ratio as a risk factor for cognitive impairment in older adults: A cross-sectional analysis of NHANES data

**DOI:** 10.1371/journal.pone.0321519

**Published:** 2025-05-05

**Authors:** Yubo Teng, Jingyi Zhang, Boxiang Yang, Qin Luo, Yu Xue, Miao Zhang

**Affiliations:** 1 Heilongjiang University of Chinese Medicine, Harbin, China; 2 The Second Hospital Affiliated of Heilongjiang University of Chinese Medicine, Harbin, China; Hospital Costa del Sol, SPAIN

## Abstract

**Background:**

Cognitive impairment is an escalating challenge in aging populations, with risk factors extending beyond traditional domains. The urine albumin-to-creatinine ratio (UACR), a marker of kidney and vascular health, has been associated with systemic dysfunction, yet its association with cognitive impairment remains underexplored. This study aims to delve into the association between UACR and cognitive function in older adults.

**Methods:**

This study conducted a cross-sectional analysis using data from the 2011–2014 National Health and Nutrition Examination Survey (NHANES), including 2,385 adults aged ≥60 years. Cognitive function was assessed using composite ‘Cognitive Scores’ derived from standardized tests. Participants were categorized into quartiles based on UACR levels. Multivariate linear regression models were used to evaluate the association between UACR and cognitive scores. Additionally, multivariate logistic regression models were used to assess the relationship between UACR and cognitive impairment, adjusting for demographic and clinical covariates. Smooth curve fitting and interaction analyses were also performed to further investigate the relationship between UACR and cognitive impairment.

**Results:**

For every 10-unit increase in UACR, cognitive scores declined by 0.019 (β = -0.019, 95% CI: -0.036 to -0.002; *P* < 0.05), and the odds of cognitive impairment increased by 2.6% (OR = 1.026, 95% CI: 1.004 to 1.048; *P* < 0.05). This trend was also observed in participants with UACR levels below 30, where for every 10-unit increase in UACR, cognitive scores decreased by 0.013 (β = -0.013, 95% CI: -0.027 to -0.001; *P* < 0.05), and the odds of cognitive impairment increased by 1.4% (OR = 1.014, 95% CI: 1.003 to 1.038; *P* < 0.05). The study also identified a nonlinear relationship, where the risk of cognitive impairment increased with rising UACR levels, but this risk plateaued at a UACR value of 12.86. Hypertension was found to be a significant factor influencing the relationship between UACR and cognitive impairment (*P* for interaction < 0.05).

**Conclusion:**

This study highlights the significant association between elevated UACR and cognitive impairment in older adults. Notably, even at lower UACR levels, increases in UACR are associated with a higher risk of cognitive impairment. These findings underscore the importance of incorporating kidney health management into strategies aimed at preventing cognitive decline. Further longitudinal studies are needed to clarify the causal relationship and explore interventions targeting UACR to preserve cognitive function.

## 1. Introduction

Cognitive function encompasses the psychological processes involved in acquiring, processing, storing, and utilizing information, including perception, attention, memory, language, and decision-making abilities [1]. As individuals age, the risk of cognitive decline increases, particularly among those over 60 years of age, with mild cognitive impairment (MCI) and dementia being more prevalent [2,3]. As the global population ages, the number of individuals at risk for cognitive impairment continues to rise, posing significant challenges to public health.

Although age-related cognitive decline is inevitable, healthy lifestyle choices and the management of certain diseases can slow the onset and progression of cognitive impairment [4,5]. Regular physical activity, cardiovascular health protection, and diets high in protein or antioxidants have been shown to effectively reduce the risk of cognitive decline [6–9]. However, biomarkers for predicting cognitive decline remain underexplored.

Chronic kidney disease (CKD) is also a significant risk factor for cognitive decline, particularly in stages 3–5 [10]. The urinary albumin-to-creatinine ratio (UACR) is a key biomarker for assessing kidney health, especially in the context of CKD [11]. Elevated UACR is closely associated with renal damage, endothelial dysfunction, and cardiovascular diseases [12,13]. While much research has focused on the relationship between UACR and cardiovascular disease [14], less attention has been given to its potential dose-response relationship with cognitive function, particularly in older populations. Given the close link between vascular health, kidney function, and brain function, UACR may provide valuable insights into the mechanisms underlying cognitive impairment.

This study aims to use data from the National Health and Nutrition Examination Survey (NHANES) to investigate the association between UACR and cognitive impairment. By leveraging this nationally representative dataset, we seek to provide new perspectives and evidence for the early detection and intervention of cognitive decline, ultimately informing strategies to mitigate cognitive decline in high-risk populations.

## 2. Materials and methods

### 2.1 Date source and participants

The data for this study were sourced from the National Health and Nutrition Examination Survey (NHANES). NHANES, conducted by the National Center for Health Statistics (NCHS) under the Centers for Disease Control and Prevention (CDC), is a nationally representative program that evaluates the health and nutritional status of adults and children in the United States by combining interviews and physical examinations to collect extensive data on disease prevalence, risk factors, and nutritional status, thereby providing critical evidence to inform public health policies and intervention strategies [15]. The study protocol for NHANES has been approved by the NCHS Research Ethics Review Board, and all participants provided written informed consent to participate. The approval numbers for the presented survey years are: Protocol #2011–17 (NHANES 2011–2012), Continuation of Protocol #2011–17 (NHANES 2013–2014) The NHANES database is publicly accessible and available for research purposes [16,17]. Further details about NHANES can be found on its official website (https://www.cdc.gov/nchs/nhanes/about_nhanes.htm).

**Fig 1:**
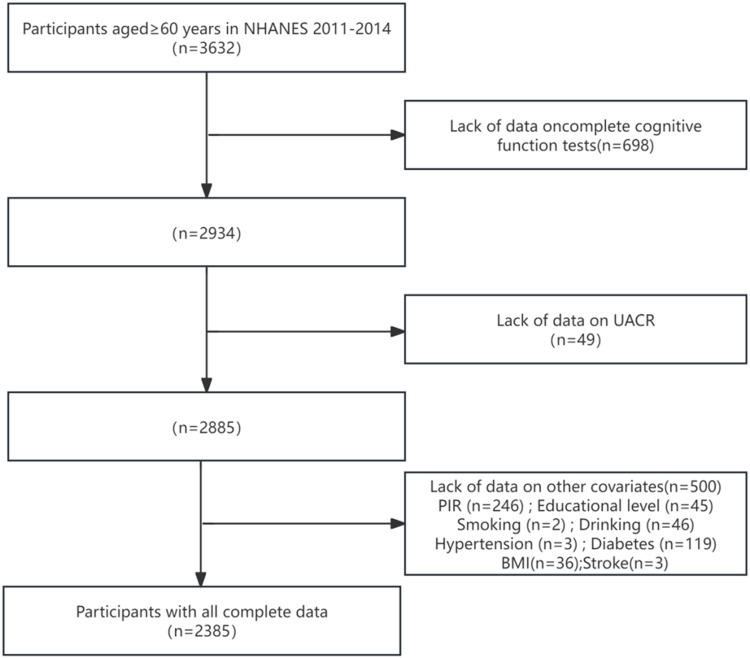
Flowchart of participants in this study.

For the present study, we included participants aged ≥60 years who underwent cognitive function assessments during the 2011–2014 NHANES cycles. Of the 3,632 eligible participants, those with incomplete cognitive assessments, missing UACR data, or other covariate information were excluded, resulting in a final analytical sample of 2,385 participants. The participant selection process is outlined in Fig 1.

### 2.2 Cognitive function assessment

Cognitive function of the included participants was evaluated using three standardized neuropsychological tests: the Consortium to Establish a Registry for Alzheimer’s Disease (CERAD) Word Learning and Recall Module, the Animal Fluency Test (AFT), and the Digit Symbol Substitution Test (DSST). The CERAD test includes three consecutive word learning trials, followed by a delayed word recall conducted after completing the AFT and DSST. This module is specifically designed to monitor cognitive function and plays a pivotal role in screening and early detection of Alzheimer’s disease (AD). Its application facilitates the early diagnosis and intervention of AD [18,19].The AFT is a widely used neuropsychological assessment tool for evaluating memory and cognitive function across diverse populations. Participants are required to name as many animals as possible within one minute, providing a quantitative measure of cognitive performance [20,21].The DSST assesses multiple cognitive domains by measuring the speed and accuracy of digit-symbol matching tasks within a set time. This test comprehensively evaluates processing speed, attention, and executive functions. Results from the DSST provide critical data for identifying cognitive abnormalities and monitoring the progression of cognitive impairment [22,23].

Based on previous studies [24,25], this study utilized a composite measure, referred to as “Cognitive Scores,” to represent participants’ cognitive performance. The Cognitive Scores were derived by combining the results of the CERAD, AFT, and DSST tests. This approach minimizes the variability introduced by using different cognitive assessment tools and ensures a more standardized evaluation of cognitive abilities. Additionally, the calculation method controlled for extreme values, thereby mitigating potential biases caused by floor or ceiling effects in cognitive assessments. “Cognitive scores” =$\sum_{1}^{3}(x-Mean)/SD$，In this study, x represents the score for a specific cognitive test, while Mean and SD denote the mean and standard deviation of the test, respectively. Given the absence of a universally accepted gold standard for diagnosing cognitive impairment, we defined cognitive impairment as having a “Cognitive Score” below the 25th percentile (*P*_25_). In this dataset, the cutoff value for cognitive impairment was *P*_25_=−1.69 [10,26].

### 2.3 Assessment of UACR

The UACR is calculated by dividing the urinary albumin concentration (μg/mL) by the urinary creatinine concentration (mg/dL). In NHANES, UACR values are standardized and reported in mg/g. Clinically, UACR is widely used for evaluating kidney function, particularly for the early detection and monitoring of proteinuria severity [27,28].The urinary albumin and creatinine concentrations for participants were obtained through processed urine samples. These samples were carefully handled and stored before being sent to the University of Minnesota Laboratory for analysis. Urinary albumin concentrations were measured using a solid-phase fluorescent immunoassay, while urinary creatinine concentrations were determined enzymatically using a Roche/Hitachi Modular P Chemistry Analyzer.

### 2.4 Covariates

The covariates included in this study were gender (male and female), age, race/ethnicity (Mexican American, other Hispanic, Non-Hispanic White, Non-Hispanic Black, and other races), educational level (below high school, high school or GED, and above high school), poverty-to-income ratio (PIR), and body mass index (BMI) classified as normal (<25 kg/m²), overweight (25 to < 30 kg/m²), and obese (≥30 kg/m²). Medical histories of hypertension, diabetes, and stroke were determined based on self-reported diagnoses by healthcare professionals. Alcohol consumption was categorized as “Never” (lifetime consumption of < 12 drinks), “Former” (≥12 drinks in a lifetime but none in the past year), or “Now” (≥12 drinks in the past year). Smoking status was defined as “Never” (lifetime smoking of < 100 cigarettes) or “Now” (≥100 cigarettes).

### 2.5 Statistical analysis

Participants were categorized into four groups based on their UACR levels. Continuous variables were expressed as mean ± standard deviation (SD), and categorical variables were presented as counts and percentages. Chi-square tests were used for categorical variables, and Kruskal-Wallis rank-sum tests were applied for continuous variables to determine p-values. Multivariate logistic regression was conducted to examine the associations between UACR, UACR quartiles, and “Cognitive Scores,” while multivariate linear regression was used to assess the relationship between UACR, UACR quartiles, and cognitive impairment. To control for confounding factors, three models were constructed: Model 1 was unadjusted, Model 2 adjusted for age, gender, and race, and Model 3 further adjusted for education level, PIR, alcohol use, smoking status, BMI, hypertension, stroke, and diabetes.

Restricted cubic spline (RCS) was used to investigate the dose-response relationship between UACR and cognitive impairment. Interaction tests between UACR and cognitive impairment were performed across subgroups stratified by age, gender, race, smoking status, alcohol consumption, history of stroke, diabetes, and hypertension, with the results illustrated using a forest plot. All analyses were conducted using EmpowerStats (version 4.1) and R software (version 4.3.3). The association between UACR and cognitive function scores was evaluated using odds ratios (OR) with 95% confidence intervals (CI), while the relationship between UACR and cognitive impairment was assessed using β coefficients and 95% CI. A *P* < 0.05 was considered statistically significant.

## 3. Results

### 3.1 Population characteristics

A total of 2,385 participants were included in the study, categorized into four groups based on UACR quartiles: Q1 (0.26–5.85), Q2 (5.85–9.76), Q3 (9.76–21.30), and Q4 (21.30–10,465.12). As shown in Table 1, all variables except race, smoking status, history of stroke, BMI, and urine creatinine exhibited statistically significant differences across the four groups (*P* < 0.05). Participants in the Q4 group were predominantly low-income females aged ≥70 years, with higher rates of diabetes and hypertension. Compared to the Q1 group, the Q4 group had significantly lower cognitive scores and a higher prevalence of cognitive impairment.

**Table 1:** Baseline characteristics of participants (N = 2385) by UACR.

	UACR categories				
Characteristic	Q1(0.26–5.85)	Q2(5.85–9.76)	Q3(9.76–21.30)	Q4(21.30–10465.12)	P
N	596	595	597	597	
Age (years) (%)					
<70	405 (67.95%)	366 (61.51%)	281 (47.07%)	259 (43.38%)	<0.001
≥70	191 (32.05%)	229 (38.49%)	316 (52.93%)	338 (56.62%)	
Gender (%)					<0.001
Male	348 (58.39%)	262 (44.03%)	264 (44.22%)	290 (48.58%)	
Female	248 (41.61%)	333 (55.97%)	333 (55.78%)	307 (51.42%)	
Race (%)					0.432
Mexican American	51 (8.56%)	58 (9.75%)	43 (7.20%)	48 (8.04%)	
Other Hispanic	57 (9.56%)	54 (9.08%)	59 (9.88%)	63 (10.55%)	
Non-Hispanic White	279 (46.81%)	295 (49.58%)	313 (52.43%)	296 (49.58%)	
Non-Hispanic Black	157 (26.34%)	128 (21.51%)	121 (20.27%)	140 (23.45%)	
Other Race	52 (8.72%)	60 (10.08%)	61 (10.22%)	50 (8.38%)	
Education level (%)					<0.001
Less Than 9th Grade	49 (8.22%)	42 (7.06%)	64 (10.72%)	73 (12.23%)	
9-11th Grade	90 (15.10%)	74 (12.44%)	73 (12.23%)	86 (14.41%)	
High School Grad/GED	115 (19.30%)	144 (24.20%)	148 (24.79%)	145 (24.29%)	
College or AA degree	174 (29.19%)	167 (28.07%)	163 (27.30%)	182 (30.49%)	
College Graduate or above	168 (28.19%)	168 (28.24%)	149 (24.96%)	111 (18.59%)	
PIR	2.85 ± 1.64	2.76 ± 1.59	2.58 ± 1.58	2.31 ± 1.55	<0.001
Alcohol use (%)					
Never	76 (12.75%)	98 (16.47%)	92 (15.41%)	101 (16.92%)	0.008
Former	168 (28.19%)	136 (22.86%)	172 (28.81%)	188 (31.49%)	
Now	352 (59.06%)	361 (60.67%)	333 (55.78%)	308 (51.59%)	
Smoking status (%)					0.186
Never	302 (50.67%)	291 (48.91%)	301 (50.42%)	328 (54.94%)	
Now	294 (49.33%)	304 (51.09%)	296 (49.58%)	269 (45.06%)	
Stroke (%)					0.312
Yes	32 (5.37%)	26 (4.37%)	33 (5.53%)	64 (10.72%)	
No	564 (94.63%)	569 (95.63%)	564 (94.47%)	533 (89.28%)	
Diabetes (%)					<0.001
Yes	95 (15.94%)	122 (20.50%)	129 (21.61%)	237 (39.70%)	
No	501 (84.06%)	473 (79.50%)	468 (78.39%)	360 (60.30%)	
Hypertension (%)					<0.001
Yes	322 (54.03%)	344 (57.82%)	362 (60.64%)	446 (74.71%)	
No	274 (45.97%)	251 (42.18%)	235 (39.36%)	151 (25.29%)	
BMI (%)					<0.218
Normal	154 (25.84%)	150 (25.21%)	179 (29.98%)	157 (26.30%)	
Overweight	226 (37.92%)	210 (35.29%)	214 (35.85%)	201 (33.67%)	
Obese	216 (36.24%)	235 (39.50%)	204 (34.17%)	239 (40.03%)	
Urine albumin (ug/ml)	4.83 ± 3.47	8.30 ± 5.69	15.35 ± 10.83	242.68 ± 657.87	<0.001
Urine creatinine (mg/dl)	9987.57 ± 6442.96	9794.87 ± 6497.51	9646.41 ± 6255.43	9289.26 ± 5654.92	0.425
CERAD	25.66 ± 5.99	25.96 ± 6.25	25.00 ± 6.58	23.58 ± 6.81	<0.001
AFT	17.40 ± 5.53	17.33 ± 5.62	16.71 ± 5.66	15.63 ± 5.06	<0.001
DSST	49.47 ± 16.69	49.09 ± 16.96	45.96 ± 17.98	41.13 ± 16.20	<0.001
Cognitive scores	0.39 ± 2.26	0.40 ± 2.38	-0.04 ± 2.49	-0.76 ± 2.35	<0.001
Cognitive impairment (%)					<0.001
Yes	108 (18.12%)	119 (20.00%)	160 (26.80%)	216 (36.18%)	
No	488 (81.88%)	476 (80.00%)	437 (73.20%)	381 (63.82%)	

Mean  ±  SD for continuous variable, number (%) for categorical variables.

### 3.2 Relationship between UACR and cognition

To evaluate the relationship between UACR and cognitive function, regression analyses were performed, incorporating both continuous UACR values and their categorical quartiles. As shown in Table 2, in the fully adjusted Model 3, each 10-unit increase in UACR was associated with a decrease of 0.019 points in cognitive scores (β = -0.019, 95% CI: -0.036 to -0.002; *P* < 0.05), and a 2.6% increase in the odds of cognitive impairment (OR = 1.026, 95% CI: 1.004 to 1.048; *P* < 0.05). Compared to the Q1 group, the Q4 group had a 0.377 lower cognitive score (β = -0.377, 95% CI: -0.600 to -0.153; *P* < 0.001) and a 78.9% higher likelihood of cognitive impairment (OR = 1.789, 95% CI: 1.304 to 2.455; *P* < 0.001).

**Table 2:** Analyzing the relationship between UACR and cognitive scores and impairment.

	Model1	Model2	Model3
Cognitive scores			
UACR # continuous	-0.057 (-0.079, -0.035)^***^	-0.033 (-0.052, -0.015)^***^	-0.019 (-0.036, -0.002)^*^
UACR categories			
Q1	0	0	0
Q2	0.013 (-0.257, 0.283)	0.007 (-0.229, 0.243)	-0.002 (-0.216, 0.212)
Q3	-0.431 (-0.701, -0.162)^**^	-0.162 (-0.400, 0.077)	-0.043 (-0.259, 0.174)
Q4	-1.156 (-1.425, -0.886)^***^	-0.691 (-0.931, -0.451)^***^	-0.377 (-0.600, -0.153)^***^
Cognitive Impairment			
UACR # continuous	1.052 (1.027, 1.077)^***^	1.035 (1.012, 1.059)^**^	1.026 (1.004, 1.048)^*^
UACR categories			
Q1	1	1	1
Q2	1.130 (0.846, 1.509)	1.172 (0.859, 1.598)	1.197 (0.862, 1.662)
Q3	1.654 (1.255, 2.180)^***^	1.478 (1.096, 1.994)^*^	1.418 (1.031, 1.952)^*^
Q4	2.562 (1.961, 3.346)^***^	2.103 (1.572, 2.813)^***^	1.789 (1.304, 2.455)^***^

UACR **#** represents 10 units of UACR; UACR **#** continuous represents a 10-unit continuous rise in UACR.

Model 1 adjusted for: none.

Model 2 adjusted for: age, gender, race.

Model 3 adjusted for: age, gender, race, education level, PIR, alcohol use, smoking status, BMI, hypertension, stroke, diabetes.

* *p* < 0.05;

** *p* < 0.01;

*** *p* < 0.001.

At baseline, more than 75% of participants had low-grade proteinuria (UACR < 30 mg/g). To further explore the relationship between UACR and cognitive function within this subgroup, regression analyses were conducted on 1936 participants with UACR < 30 mg/g, including both continuous UACR values and its categorical quartiles. As shown in Table 3, in the fully adjusted Model 3, each 10-unit higher in UACR was associated with a 0.013-point lower in cognitive scores (β = -0.013, 95% CI: -0.027 to -0.001; *P* < 0.05) and a 1.4% higher in the odds of cognitive impairment (OR = 1.014, 95% CI: 1.003 to 1.038; *P* < 0.05). Compared to the Q1 group, the Q4 group showed a 0.228-point lower in cognitive scores (β = -0.228, 95% CI: -0.472 to -0.124; *P* < 0.05) and a 49.2% higher likelihood of cognitive impairment (OR = 1.492, 95% CI: 1.041 to 2.137; *P* < 0.001).

**Table 3:** Analyzing the relationship between UACR (<30) and cognitive scores and impairment.

	Model1	Model2	Model3
Cognitive scores	β (95% CI)/OR (95% CI)	β (95% CI)/OR (95% CI)	β (95% CI)/OR (95% CI)
UACR # continuous	-0.042 (-0.059, -0.025) ^***^	-0.023 (-0.038, -0.007) ^***^	-0.013 (-0.027, -0.001) ^*^
UACR categories			
Q1	0	0	0
Q2	0.166 (-0.134, 0.466)	0.004 (-0.257, 0.264)	-0.063 (-0.301, 0.174)
Q3	-0.158 (-0.458, 0.142)	-0.115 (-0.378, 0.147)	-0.099 (-0.339, 0.140)
Q4	-0.594 (-0.894, -0.293) ^***^	-0.346 (-0.613, -0.080) ^*^	-0.228 (-0.472, -0.124) ^*^
Cognitive Impairment			
UACR # continuous	1.034 (1.138, 1.582) ^*^	1.021 (1.004, 1.451) ^*^	1.014 (1.003, 1.038) ^*^
UACR categories			
Q1	1	1	1
Q2	1.018 (0.735, 1.409)	1.155 (0.814, 1.638)	1.27 (0.875, 1.831)
Q3	1.332 (0.974, 1.823)	1.337 (0.951, 1.878)	1.40 (0.974, 2.000)
Q4	1.750 (1.290, 2.372) ^***^	1.554 (1.109, 2.178) ^*^	1.492 (1.041, 2.137) ^*^

UACR **#** represents 10 units of UACR；UACR **#** continuous represents a 10-unit continuous rise in UACR.

Model 1 adjusted for: none.

Model 2 adjusted for: age, gender, race.

Model 3 adjusted for: age, gender, race, education level, PIR, alcohol use, smoking status, BMI, hypertension, stroke, diabetes.

* *p* < 0.05;

** *p* < 0.01;

*** *p* < 0.001.

**Fig 2:**
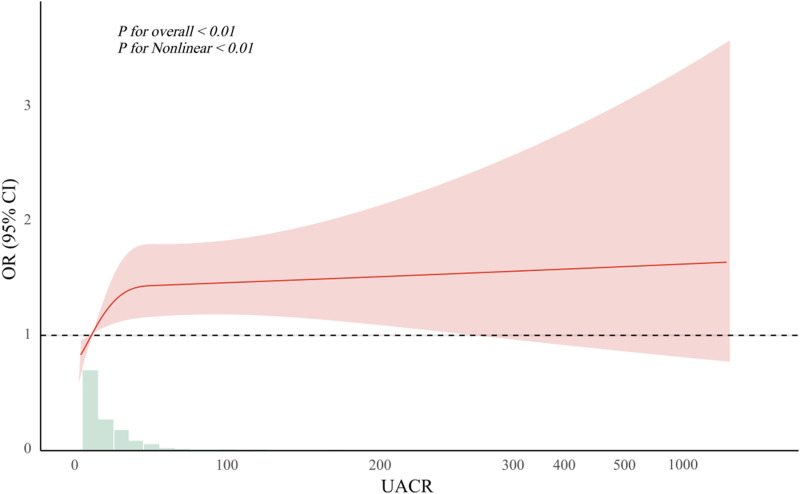
Nonlinear relationship between UACR and cognitive impairment.

In a fully adjusted model accounting for potential confounders, RCS analysis revealed a significant nonlinear relationship between UACR and cognitive impairment (*P* for nonlinearity < 0.01) (Fig 2). The risk of cognitive impairment increased with rising UACR levels, plateauing when UACR reached 12.86 (OR: 1.052, 95% CI: 1.018–1.088; *P* < 0.01) (Fig 2, Table 4). The RCS analysis further indicated that UACR values were predominantly clustered in the lower range, emphasizing the need for monitoring individuals with low UACR levels.

**Table 4:** Threshold effect analysis of UACR and cognitive impairment.

UACR	OR (95%CI)	*P*-Value
<12.86	1.052(1.018,1.088)	0.003
≥12.86	1.001(0.999,1.003)	0.38
*P* for likelihood ratio test		0.004

### 3.3 Subgroup analysis

**Fig 3:**
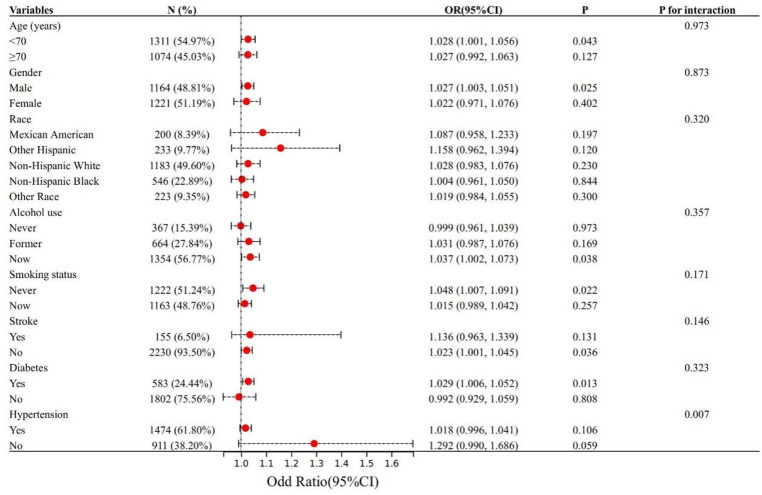
Forest plot between UCAR and cognitive impairment.

To evaluate the reliability of the association between UACR and cognitive impairment, subgroup analyses were conducted based on factors including age, gender, race, alcohol consumption, smoking status, hypertension, diabetes, and stroke. Additionally, interaction effects among these subgroups were assessed. As shown in Fig 3, a positive association between UACR and cognitive impairment was observed across all subgroups; however, in some subgroups, the association did not reach statistical significance (*P* > 0.05). Notably, a significant interaction effect was identified in the subgroup with hypertension (*P* for interaction < 0.05), suggesting that hypertension may play a key role in modulating the relationship between UACR and cognitive impairment.

## 4. Discussion

This study utilized cross-sectional data from 2385 elderly individuals (aged ≥60 years) in the NHANES database to investigate the relationship between UACR and cognitive function. We found a significant negative correlation between UACR levels and cognitive performance, with higher UACR associated with higher odds of cognitive impairment. Notably, this trend persisted even when UACR levels were below 30 mg/g. Furthermore, RCS and threshold effect analyses revealed that the odds of cognitive impairment increased with rising UACR levels, stabilizing once UACR reached 12.86 mg/g. Subgroup analyses confirmed the robustness of this association across various populations and clinical characteristics, suggesting that hypertension may play a modulating role in the relationship between UACR and cognitive decline.

Unlike prior studies, which predominantly focused on the relationship between CKD stages and cognitive function, our study not only corroborated the positive association between UACR and cognitive impairment but also explored this relationship within the low-proteinuria cohort (UACR <30 mg/g). Previous NHANES studies have primarily investigated CKD stages in relation to cognitive function, with limited focus on the direct impact of UACR [10,29]. Additionally, a prospective cohort study from the Atherosclerosis Risk in Communities (ARIC) study found that elevated UACR was associated with an increased risk of cognitive impairment [30], which is consistent with our findings. However, our study adds a novel perspective by providing evidence for the association between UACR and cognitive impairment in populations with low proteinuria, thereby extending current knowledge in this field.

Historically, research has primarily focused on the relationship between UACR and cardiovascular diseases, with elevated UACR linked to endothelial dysfunction, arterial thickening, and increased arterial stiffness [31,32]. These cardiovascular alterations not only impact heart health but may also lead to reduced cerebral blood flow and heightened neuroinflammation, ultimately impairing cognitive function [33,34]. Additionally, arterial thickening and stiffness can contribute to small vessel disease in the brain, further exacerbating cognitive decline [35–37]. Persistent proteinuria, a hallmark of elevated UACR, may damage cognitive function through multiple mechanisms, including oxidative stress, neurotransmitter imbalances, hemodynamic changes, and neurotoxicity [38]. Previous studies have shown that both persistent and progressive proteinuria are closely associated with an increased risk of cognitive decline, particularly vascular dementia (VaD) and other forms of dementia [39]. Another study highlighted that increased urinary albumin is linked to higher risks of MCI and dementia, likely mediated through mechanisms such as hippocampal atrophy, neuroinflammation, oxidative stress, and disruption of the blood-brain barrier (BBB) [40].

Hypertension plays a pivotal role in modulating the relationship between UACR and cognitive impairment. Not only is hypertension a risk factor for elevated UACR, but increased UACR also correlates with an increased risk of hypertension, creating a complex bidirectional relationship [41]. Hypertension is also an independent risk factor for cognitive dysfunction [42]. Through mechanisms such as microvascular damage, BBB disruption, atherosclerosis, and endothelial dysfunction, hypertension exacerbates cognitive decline. Hypertension accelerates brain microvascular injury via BBB disruption, increasing the risk of VaD and other dementias [43]. Moreover, hypertension promotes atherosclerosis, which impairs cerebral blood flow and accelerates cognitive decline, particularly through vascular changes such as endothelial dysfunction [44]. Chronic hypertension may also accelerate cognitive decline through mechanisms like neuroinflammation and microvascular damage, particularly in the elderly [45].

Despite these important findings, several limitations must be acknowledged. First, the cross-sectional design of this study using NHANES data precludes any causal inference, thus limiting our ability to draw direct cause-and-effect relationships between UACR levels and cognitive impairment. Second, extreme values observed in the Q4 group may impact the accuracy and robustness of the analysis. There is no standardized method for handling extreme values in NHANES data, and this could affect the results. Future studies could employ longitudinal data or more precise experimental designs to validate the relationship between UACR and cognitive impairment and further explore its underlying biological mechanisms. Additionally, future research could consider using more appropriate data processing methods, such as data transformation or the removal of outliers, to improve the reliability of the findings. These efforts would contribute to a clearer understanding of the association between UACR and cognitive function.

## 5 Conclusion

This study investigated the relationship between UACR and cognitive function in 2,385 elderly Individuals aged ≥60 years from the United States. Our findings demonstrated a significant negative correlation between UACR and cognitive impairment, with this association remaining evident even in the low-proteinuria group (UACR <30 mg/g). While this study provides new evidence for the potential of UACR as a biomarker for cognitive dysfunction risk, the cross-sectional design limits causal inference. Future studies should employ longitudinal data to further validate this association and explore the underlying mechanisms.
